# The determinants of burnout and professional turnover intentions among Canadian physicians: application of the job demands-resources model

**DOI:** 10.1186/s12913-021-06981-5

**Published:** 2021-09-20

**Authors:** Denis Chênevert, Steven Kilroy, Kevin Johnson, Pierre-Luc Fournier

**Affiliations:** 1 Department of HR Management, HEC, Montréal, Québec Canada; 2 Department of HR Studies, Tilburg University, Tilburg, The Netherlands; 3 Management School, Université de Sherbrooke, Montréal, Québec Canada

## Abstract

**Background:**

Burnout among physicians is growing at an exponential rate and many are leaving the profession. Nevertheless, the specific antecedents and intermediary stages involved in predicting their professional turnover intentions are not fully clear.

**Purpose:**

We apply the Job Demands-Resources model and investigate an innovative model which predicts physician burnout and its ultimate consequences on professional turnover intentions.

**Methodology/approach:**

Structural equation modeling was used on cross-sectional survey data from a sample of 407 Canadian physicians.

**Results/conclusions:**

Job demands (work stress, work overload, and work-family conflict) and job resources (patient recognition and meaning at work) influence intention to leave the profession through a two stage health-impairment and motivational process related to health problems and professional commitment, respectively.

**Practical implications:**

This study identifies key job resources and job demands which predict physician burnout and professional turnover intentions thereby pinpointing which levers managers can use improve their health and retain them in the profession.

## Introduction

The latest studies suggest that physician work stress and emotional exhaustion is on the rise and reported to be significantly higher than that of the general population [39]. Excessive work overload, continuous change, stress and work family balance issues impair physicians’ health and indirectly the quality of care provided [31]. This overload situation and inability to effectively administer optimal patient care, can cause stress and burnout, thereby prompting physicians to consider leaving the profession [26, 32, 47]. Physician shortages have become a pervasive issue worldwide [48]. Given the significant shortage of physicians and since exit from the profession due to burnout is one of the primary reasons in this regard [32], it apposite to examine intention to leave the profession as a withdrawal mechanism from experienced burnout. The job demands-resources model (JD-R) [12], purports that job demands are the aggravating factors for burnout while job resources are expected to alleviate burnout. A recent systematic review has revealed that there has been a paucity of sophisticated models applied to understand physician burnout and its nomological network [46]. In particular, little is known about the distinctive role of job demands and job resources, and how they transpire into heightened levels of burnout and intention to leave the medical profession. We aim to address this void and formulate the general research question: What is the role of burnout in the relationship between the job demands and job resources linked to physicians’ work and their intention to leave the medical profession?

### Theory

#### Conceptual model and hypotheses development

Burnout is a workplace phenomenon characterized by the three symptoms of emotional exhaustion, depersonalization and reduced professional efficacy [45]. Emotional exhaustion refers to a state of mental weariness whereby employees are depleted of their emotional and energetic resources while depersonalization refers to a process whereby employees develop an uncaring or cynical attitude towards their job, their performance and those associated with the job (e.g. patients and coworkers) [28]. Reduced professional efficacy refers toa decline in one’s feelings of competence on the job and while initially representing a dimension of burnout, scholars now tend to view the concept as more akin to a personality trait rather than a core dimension of burnout [10]. The notion that emotional exhaustion and depersonalization comprise the core symptoms of burnout has been substantiated by recent empirical evidence testing the factorial structure of the burnout measure leading the authors to conclude that reduced professional accomplishment “plays a divergent role in the burnout phenomenon” (p222) [11]. Therefore, for the present study we will focus on a two-dimensional definition of burnout which captures emotional exhaustion and depersonalization, which is also consistent with the approach of the founding theorists of the JD-R model [12].

**Fig. 1:**
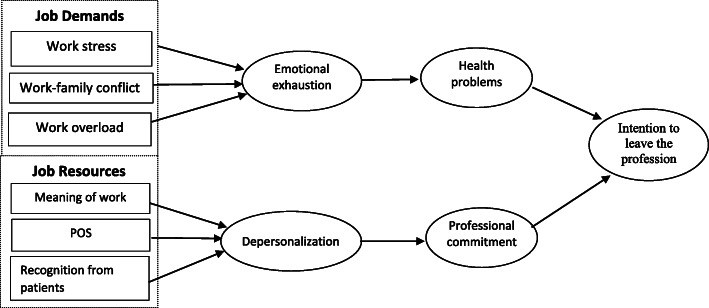
Theoretical model

The initial conception of the JD-R model posits that there are two distinct yet related processes at play in the development of burnout [12]. Job demands activate a health-impairment process through emotional exhaustion, whereas job resources activate a motivational process through depersonalization/disengagement [12]. In line with these propositions, we propose a comprehensive model (See Fig. 1) which links job demands and job resources to health problems and professional commitment, respectively, via the health impairment process i.e. emotional exhaustion, and via the motivational process i.e. depersonalization, and in turn chart their effects on the intention to leave the medical profession. We now turn to a discussion of the key direct relationships in the proposed serial mediation model (See Fig. 1), which make up our hypotheses.

#### The health impairment process: job demands and emotional exhaustion

Physicians work long work hours and in emergency situations that may affect their stress and ability to achieve work-life balance [33]. Indeed, stress, excessive workload, and a difficult work-life balance are among the most pressing job demands for physicians [15], and can result in them losing sight of the inspiring and rewarding moments of care provision [31]. In line with the JD-R model, burnout represents an outcome of such job demands and stress factors whereby individual’s energetic resources become depleted over time [22]. In an effort to deal with job demands and protect one’s valued energetic resources, other resources have to be invested (e.g. more time and effort, sacrifice), which carries the risk of burnout and consequently long-term absence. Supporting this proposition, Lee et al. [24] demonstrated that job demands are positively linked to emotional exhaustion among physicians. Furthermore, stress has also been associated with higher burnout over time among physicians [33]. Therefore, we propose the following hypotheses:

- *H1: a) Stress, b) quantitative overload and c) work-family conflicts are positively linked to emotional exhaustion.*

#### Emotional exhaustion and health problems

Job demands such as work overload, stress and work-family conflicts are associated with considerable health impairment effects. However, on the basis of the relationship between job demands and health, we argue that these effects are indirect. Chronic stress from work-related job demands imposes a resource depletion process according to the JD-R model [22] which ultimately transpires into burnout. Considering that burnout is studied as a process proceeding through various stages from stressful job demands to serious physical symptoms and despair [47], a higher level of emotional exhaustion indicates a more critical progression of the resource depletion process, thus a lower and more critical level of remaining resources to face job demands. Toward an end state, when job demands are still apparent, but job resources are decreased and emotional exhaustion is therefore high, comes health problems and physical symptoms [44]. Williams et al.’s [46] recent systematic review of the relationships between emotional exhaustion and health problems showed that only four studies out of five have empirically addressed this important issue albeit most evidence substantiates the detrimental effects of emotional exhaustion for employees’ health. Therefore, we hypothesize that:

- *H2: Emotional exhaustion is positively associated with health problems.*

#### The motivational process: job resources and depersonalization

In the motivational process of the JD-R model, job resources facilitate engagement, directly reduce burnout and can buffer against the deleterious effects of job demands on burnout [12]. Job resources are especially predictive of depersonalization according to the JD-R model [12, 19]. The central role of resources in curbing burnout can be explained by the fact that job resources represent intrinsic and extrinsic sources of motivation for an individual and are instrumental in helping them achieve work goals. Conversely, a lack of job resources at work is a potential threat which requires one’s coping mechanisms and one coping response by physicians can be detachment from work i.e. depersonalization [33]. Moreover, consistent with social exchange theory, if employees feel that they continuously contribute more to the employment relationships than they receive, this violates the reciprocity principle causing them to feel stress and burnout [42]. Vultée et al. [43] found that physicians experience a sense of failure, frustration and thus a decreased motivation when their personal performance standards are hindered by a lack of support, recognition, and meaning of work. Meaning is regarded as a psychological job resource given its centrality to the intrinsic motivation of employees [19] and is especially important for minimizing disengagement among physicians [31 29]. Social support and recognition from patients represent job resources which emanate from the social environment [19] and are also believed to play an instrumental role in activating the engagement and motivation of physicians [31]. Lee et al. [24] showed the positive effects of job resources such as support on depersonalization levels. A better understanding of depersonalization as a potential mediator toward disengagement may enable precision to our understanding of the role of various resources in predicting depersonalization and its consequences [24]. Therefore, we propose the following hypotheses:

- H3 a) Organizational support, b) recognition from patients and c) meaning of work are negatively linked to depersonalization.

#### Depersonalization and professional commitment

Meyer and Herscovitch [29] contend that professional commitment constitutes a psychological state that leads employees to want to maintain their occupational identification. This state is explained by mechanisms linked to identification, intrinsic motivation and sharing of values; mechanisms that can explain the ties between the job resources of the JD-R model and professional commitment through depersonalization. First, the lack of job resources that foster personal growth may lower intrinsic motivation and prompt disengagement from an unsatisfactory occupational activity. For physicians, this may translate into depersonalization specifically towards patients [24]. This distancing within physicians’ relational sphere initiates a process of erosion of professional commitment. Lastly, from an empirical standpoint, the meta-analysis by Lee et al. [23] demonstrates that depersonalization is negatively linked with professional commitment (rc = −.37). Therefore, we propose the following hypothesis:

- *H4: Depersonalization is negatively associated with professional commitment.*

#### Intention to leave the profession

The decision to leave the profession for physicians experiencing burnout is mainly viewed as resulting from a long and difficult deliberation process given their high levels of professional attachment [38]. *Fishbein* and *Ajzen’s* [14] theory of reasoned action stipulates that behavioral intention is the immediate antecedent of actual behavior. Accordingly, intention to leave the profession is considered a fairly robust determinant of one’s decision to leave. Wallace et al. [44] contend that burnout is a determining factor with regard to physicians’ intention to leave their job or even the occupation. The authors found that 50% of physicians think about leaving their job each week and 30% consider abandoning medicine altogether. However, the relationship between burnout and intention to leave the profession may not necessarily be direct: it is likely to be explained by an attitudinal change towards the profession [7].

In the model developed by Rhodes and Doering [35], intention to change careers is directly influenced by one’s level of satisfaction with their career. The meta-analysis by Lee et al. [23] demonstrates that professional commitment is a robust determinant of intention to leave the profession (rc = .62). Given the high degree of professional commitment that physicians possess, it is plausible to suggest that it represents a salient predictor of departing the profession. Although Rhodes and Doering’s [35] model of career change considers various personal factors (e.g. seniority, work-family conflict), it overlooks health problems, which may also motivate physicians to leave their profession [32]. The JD-R model views burnout and its health consequences as resource depleting [12]. One way of attempting to conserve any remaining resources or recoup lost resources is by reducing activity. Therefore, thinking about or planning to leave ones occupation is a coping mechanism to reduce health problems by creating a psychological release valve of sorts [46]. Thus, the arguments and evidence above inform the following hypotheses:

- *H5: Professional commitment is negatively associated with intention to leave the profession.*
- *H6: Health problems are positively associated with intention to leave the profession.*

## Methods

### Sampling and procedure

The study was conducted among 407 Canadian physicians divided into two samples: one comprising 151 general practitioners and specialists in a rural area containing a total of 500 physicians (response rate of 30%) and the other sample comprising 256 laboratory physician specialists in the province of Québec, Canada out of a population of 812 (response rate of 31.5%). A questionnaire was distributed online to physicians through the hospital for the first sample and through the laboratory specialist’s physician federation for the second sample. The final sample consisted of 77 general practitioners and 330 physician specialists, of whom 54.4% were male and whose average age was 47.34 (SD = 10.801) years. Given the length of the questionnaire, we had to restrict the number of statements per variable and thus frequently use an abbreviated form of the focal measures.

### Measurement of variables

Unless stated otherwise, items were rated on a continuum ranging from disagree completely (1) to agree completely 7). Where abbreviated scales were used, the items with the highest factor loadings from the original scales were chosen.

#### Job resources

Organizational support was measured using three statements from the instrument of Eisenberger et al. [13] (e.g. “I can count on help from my organization if I have a problem at work”). Meaning was measured using three statements taken from the scale of Spreitzer [41]. (e.g. “The work I do is very important to me”). Recognition from patients was measured using the three item scale developed by Jourdain and Chênevert [19] consisting of three items (e.g. “The patients often express their satisfaction with the services that I provide to them”).

#### Job demands

Work stress was evaluated by three items from the instrument developed by Rizzo et al. [36]. This measurement scale is designed to capture how frequently, in the previous 12 months, a person felt nervous or frustrated with daily problems at work and felt as if they could not overcome difficulties at work (e.g. “How many times did you feel nervous and stressed at work?”). Four items for work-family conflict were taken from the scale developed by Gutek et al. [16], and extended by Carlson and Perrewé [9] (e.g. “My job prevents me from spending as much time as I would like on my family life). To measure work overload, the scale designed by Caplan et al. [8] was used (e.g. “I regularly feel overloaded by my work”).

#### Endogenous variables

Burnout was measured with the Maslach Burnout Inventory-Human Services Survey (MBI-HSS) [27]. Specifically, emotional exhaustion (e.g. I feel emotionally drained by my work) and depersonalization (e.g. I have become tougher and less sensitive toward people since I started working with patients) were measured with seven and three items respectively. Participating physicians were asked to indicate the frequency of symptoms felt in the past 12 months, on a continuum ranging from never (1) to a few times a month (4) to every day (7). Regarding professional commitment, three items selected based on their factor loading were taken from the instrument of Meyer et al. [30] (e.g. I am proud to be a physician). Health problems were evaluated based on those included in Becks’ [3] “Depression Inventory”; three items were formulated to measure these problems on a frequency scale rather than according to the severity of the symptom. Accordingly, statements refer to sleep, appetite, and health problems (backaches, migraines, and respiratory and digestive problems). Physicians were asked to indicate how often they experienced the symptoms in the past 12 months, on a continuum ranging from never (1) to a few times a month (4) to every day (7). Lastly, three statements that refer to intention to leave the profession were developed by Meyer et al. [30] and adapted to an agree–disagree scale (e.g. “It is possible that I quit my medical specialization before next year”).

#### Control variables

We controlled for sex, age, and specialization. Age was measured using a continuing variable, sex was coded 1 = female and 2 = male, and specialization was divided into two categories: 1 = specialist and 2 = general practitioner.

### Data analysis

The overall hypothesized model was tested using structural equation modeling (SEM) with Amos version 24. As Anderson and Gerbing [1] recommend, we analyzed the quality of the fit of the measurement model first using confirmatory factor analysis (CFA), and then validated the structural model for hypotheses testing. The quality of fit of the model was evaluated based on commonly used indices such as the: a) chi-square test, b) comparative fit index (CFI); c) Tucker-Lewis index (TLI); d) incremental fit index (IFI); and d) root mean square error of approximation RMSEA [4]. It is commonly accepted that the model exhibits a good quality of fit when the RMSEA is situated below 0.08, and the CFI, TLI and IFI are between 0.90 and 1 [4].

## Results

### Model development phase

The fit of the measurement model was evaluated using first-order confirmatory factor analysis (CFA). Table 1 presents the results, which suggest a good level of fit between the theoretical model and the data [χ^2^ (647) = 1503.95; *p* < 0.001; CFI = 0.93; IFI = 0.93; TLI = 0.91, RMSEA = 0.057]. This model fit is superior to all the other models tested. When job resources (organizational support, recognition by patients and meaning of work) or job demands (stress at work, work-family conflict and overload) are grouped, a significant weaker fit is found. The same results are observed regarding the various dimensions of burnout, professional commitment, and health problems.

**Table 1:** Fit indices of the confirmatory factor analysis measurement model

Model	χ^2^	*df*	Δ *χ*^2^	Δ *df*	*CFI*	*IFI*	*RMSEA*
1. 11-factor theoretical model	1503.95***	647	–	–	0.93	0.93	0.057
2. Combining organizational support with recognition from patients	2218.92***	657	714.97***	10	0.86	0.87	0.077
3. Combining organizational support, recognition from patients and meaning of work	2890.02***	666	1386.07***	19	0.81	0.81	0.091
4. Combining stress at work and work/life conflict	1846.69***	657	342.74***	10	0.90	0.89	0.067
5. Combining stress at work, work-life conflict and quantitative overload	2035.76***	666	531.81***	19	0.88	0.88	0.071
6. Combining depersonalization and emotional exhaustion	1835.24***	657	331.29***	10	0.90	0.90	0.066
7. Combining depersonalization, emotional exhaustion and professional commitment	2516.93***	666	1012.98***	19	0.84	0.84	0.083
8. Combining depersonalization, emotional exhaustion, professional commitment and health problems	2579.33***	674	1075.38***	27	83	83	0.083

*Note*. *N* = 407. *CFI* = comparative fit index; *IFI* = incremental fit index; *RMSEA* = root mean square error of approximation

***p < 0,001

### Common method Bias

Survey-based data collection poses common method bias (CMV) risks. However, we tested for this potential bias by performing a CFA using the latent factor test [34], which introduces a single latent factor into the initial measurement model. The factor loadings remained significant and the model fit didn’t improve by incorporating this latent factor, indicating that CMV did not pose a threat to the data.

### Correlations

#### Descriptive statistics and correlations between the latent variables

Table 2 presents the means, standard deviations and correlations between the focal variables.

**Table 2:** Descriptive statistics and correlations for the study variables

Variables			1	2	3	4	5	6	7	8	9	10	11	12	13	14	15
	Mean	S. D.															
1. Gender	–	–	–														
2. Age	47.34	10.80	−.25**	–													
3. Experience	16.92	10.81	−.16**	.91**	–												
4. Specialization	–	–	.29**	−.19**	−.06	–											
5. Perceived organizational support	3.29	1.33	−.07	−.01	−.04	−.02	(.87)										
6. Recognition from patients	5.26	1.29	.02	−.09	.05	.03	.18**	(.95)									
7. Meaning of work	6.09	0.79	−.06	.12*	.13*	−.14**	.08	.24**	(87)								
8. Work stress	3.43	1.33	.03	−.15*	−.17**	−.15**	−.29**	−.15**	−.07	(.84)							
9. Work-family conflict	4.62	1.36	.13**	−.22**	−.21**	.10*	−.24**	−.10*	−.05	.55**	(.85)						
10. Work overload	5.28	1.16	.19**	−.24**	−.20**	.12*	−.27**	−.12*	.03	.57**	.77**	(.84)					
11. Depersonalization	2.01	1.11	.00	−.18**	−.19**	.01	−.16**	−.29**	−.26**	.43**	.31**	.28**	(.78)				
12. Emotional exhaustion	3.30	1.37	.06	−.08	−.11*	−.16**	−.28**	−.11*	−.04	.84**	.64**	.61**	.43**	(.94)			
13. Professional commitment	5.72	1.21	−.17**	.28**	.20**	−.25**	.31**	.27**	.47**	−.33**	−.39**	−.30**	−.41**	−.35**	(.81)		
14. Health problems	2.36	1.07	.11*	−.05	−.07	−.18**	−.17**	−.03	−.10*	.60**	.48**	.37**	.29**	.63**	−.29**	(.67)	
15. Intention to leave the profession	2.76	1.57	−.09	−.20**	−.13**	.17**	−.37**	−.22**	−.30**	.41**	.44**	.37**	.35**	.44**	−.72**	.36**	(.91)

*Note: Ns = 407. Alpha coefficients are reported within parentheses along the diagonal*

* p < .05

** p < .01

### Analysis of the structural equation model

**Fig. 2:**
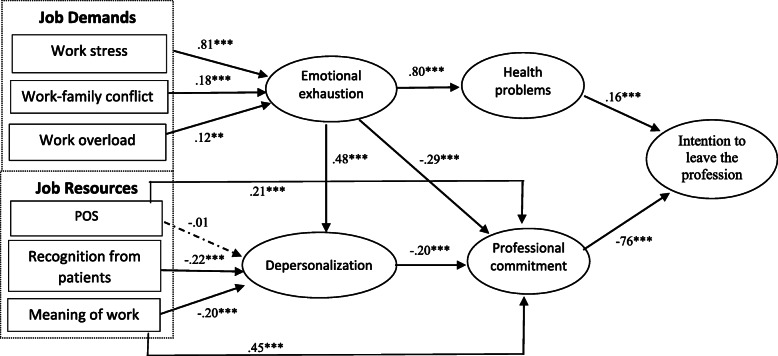
Completely standardized path coefficients for the final structural model

SEM was used to test the proposed hypotheses. The model fit of the proposed structural model is satisfactory (χ^2^(678) = 1789.52 *p* < .001; CFI = .903; IFI = .903; TLI = .900; RMSEA = .064). The variance explained for each endogenous variable is 21.5% for depersonalization, 88.4% for emotional exhaustion, 65.4% for health problems, 27.6% for professional commitment and 67.5% for intention to leave the profession. We compared this model with a series of alternative nested models. First, adding the link between emotional exhaustion and professional commitment improved the fit of the model χ^2^(1) = 20.80, *p* < .001. Second, adding the link between emotional exhaustion and depersonalization also improves model fit χ^2^(1) = 82.30, *p* < .001. In addition, adding the link between meaning of work and professional commitment improves model fit significantly χ^2^(1) = 74.97, *p* < .001. Finally, adding the link between organizational support (POS) and professional commitment also significantly improves model fit χ^2^(1) = 28.11, *p* < .001. Based on the improved model fit, we added these aforementioned links in our revised structural model as depicted in Fig. 2. The other possible links didn’t improve model fit. Lastly, adding the control variables (age, gender and work experience) did not significantly affect the results presented in Fig. 2 and they are not significant.

### Hypothesis tests

As Fig. 2 illustrates, job demands including stress at work, work overload and work-family conflict are all linked to emotional exhaustion (β = .81; *p* < 0.001; β = .12; *p* < 0.01; β = .18; *p* < 0.001), supporting hypotheses 1 a, b and c. Emotional exhaustion is positively linked to health problems (β = .81; *p* < 0.001), whereas depersonalization is negatively linked to professional commitment (β = −.20; *p* < 0.001), supporting hypotheses 2 and 4. Organizational support is not significantly linked to depersonalization (β = −.01; N.S.), refuting hypothesis 3a. However, the job resources recognition from patients and meaning are negatively linked to depersonalization (β = −.25; p < 0.001; β = −.26; p < 0.001), which affirms hypotheses 3b and 3c. Lastly, health problems are positively linked (β = .16; p < 0.001), whereas professional commitment is negatively linked (β = −.76; p < 0.001) to intention to leave the profession, affirming hypotheses 5 and 6. The links in the final model also indicate that emotional exhaustion is negatively related to professional commitment (β = − 29; *p* < .001), that emotional exhaustion is positively related to depersonalization (β = .48; p < .001), and meaning and organizational support are both positively related to professional commitment (β = .46; p < .001 and β = .21; p < .001).

### Test of the mediation hypotheses

We estimated the degree of significance of the indirect effect of depersonalization and emotional exhaustion on intention to leave the profession using the bootstrap approach. Specifically, we generated 5000 samples to attain 95% bias-corrected confidence intervals (CIs) [25]. We observed a positive indirect effect (0.15; 95% CI = 0.03–0.28) of depersonalization on intention to leave the profession via professional commitment. We also observed a positive indirect effect (0.42; 95% CI = 0.33–0.51) of emotional exhaustion on intention to leave the profession via health problems.

We did the same test on the indirect effects between job demands, job resources and the respective outcomes (health problems and professional commitment) via emotional exhaustion and depersonalization. The results suggest a positive indirect effect (0.59, 95% CI = 0.58–0.81) of work stress on health problems via emotional exhaustion and a positive indirect effect (0.08, 95% CI = 0.00–0.17) of work overload on health problems via emotional exhaustion. The results suggest also the same positive indirect effect (0.13, 95% CI = 0.07–0.22) of work-family conflict on health problems via emotional exhaustion. In the case of the job resources, the results suggest a positive indirect effect (0.04, 95% CI = 0.01–0.09) of patients’ recognition on professional commitment via depersonalization and a positive indirect effect (0.04, 95% CI = 0.01–0.08) of meaning of work on professional commitment via depersonalization.

## Discussion

The aim of the present study was to test the initial conception of the JD-R model linking specific job demands and job resources to the professional turnover intentions of physicians and investigate the health impairment and motivational pathways underlying these relationships. Our study revealed that two out of the three job resources investigated (meaning and recognition from patients) are negatively associated with depersonalization. However, support from the organization did not exert a significant impact suggesting that physicians value the meaning of work and recognition from patients more than support from the organization more generally. The time that physicians dedicate to tasks of significance represents a very strong alleviant of burnout [31]. Experiencing meaning at work is the ‘raison d’être’ for physicians, perhaps explaining why it not only helps protect against depersonalization, but also exerts a powerful direct effect on professional commitment [18]. Similarly, a ‘healthy work environment’ for physicians is one in which they receive recognition, which research suggests can increase their overall satisfaction and alleviate burnout [31]. The reason for the non-significant role of organizational support may relate to the context. Most Canadian physicians are self-employed workers. This employment status conducive to autonomy and relational distancing with the authorities that govern healthcare institutions, can reduce the role of organizational support in the burnout process. Contrary to other healthcare professionals subject to subordination rules, physicians direct their medical practice as if it were a small private business within a hospital. Thus, they are probably more receptive to more tangible support from their colleagues. Interestingly, however, organizational support did impact professional commitment directly attesting that while not instrumental for burnout, is indeed important for driving professional oriented outcomes.

Regarding job demands, stress is a strong precursor of emotional exhaustion. This finding is consistent with the JD-R model and in particular the recent conceptualization of physician burnout as a ‘loss spiral’ starting with stress ultimately culminating into burnout and health problems [46]. Compounding this stress is the harmful effect of a sense of work overload and the difficulty associated with maintaining a work-life balance [15, 31], as these factors were also empirically linked to emotional exhaustion in the present study.

Regarding the nature of the relationship between burnout and intention to leave the profession, the results indicate that both emotional exhaustion and depersonalization are linked to health problems and to professional commitment respectively, which in turn are associated with intention to leave the profession. However, emotional exhaustion which emanates from job demands has a greater indirect effect on intention to leave the profession (.42, *p* < .001) than does depersonalization (.15, p < .001). This finding is consistent with the notion that the energetic process involved in the development of burnout plays a pivotal role in the professional turnover intensions of health care professionals [19] although the findings suggest that emotional exhaustion influences professional turnover intentions indirectly through depersonalization too. The observed stronger correlation that existed between emotional exhaustion and health problems, and between depersonalization and professional commitment, largely confirm the health impairment and motivational pathways of the JD-R model.

Contrary to Bakker and Demerouti [2], our results suggest that job resources are not directly linked to emotional exhaustion or health problems. However, emotional exhaustion does influence the motivational process by influencing depersonalization and professional commitment. This finding largely confirm the theoretical postulations inherent to conservation of resources (COR) theory (the leading theory used to explain relationships in the JD-R model), that ‘resource loss’ is much more salient than resource gain in predicting burnout and the subsequent outcomes that ensue from the ‘loss spiralling’ process [17]. Indeed, demands cause a loss of important resources for employees while insufficient resources only reduce their capacity to increase their pool of existing resources. This finding also speaks to a criticism of the JD-R model which pinpoints that the health-impairment process and the motivational process may not be independent but represent two sides of the same coin [37]. While we don’t find support for the notion that job resources impact the health-impairment process, we do elucidate the power of job demands in this context as they impact both the health-impairment and motivational process simultaneously. However, the type of job resources chosen for the present investigation are by means exhaustive and therefore may explain these findings. Moreover, a potential explanation is that the JD-R model has evolved to now regard burnout (emotional exhaustion and depersonalization) as representing the health impairment process and engagement (vigour and dedication) to represent the motivational pathway [37].

As a whole, the present investigation identifies key job demands and job resources that kick-start the professional turnover intentions of physicians and pinpoints the underlying mechanisms involved. A recent systematic review has noted a paucity of theoretical models applied to understand physician burnout and elucidate that the majority of studies to date have used regression analysis (whereby antecedents and outcomes are often mixed) which limits our understanding of the potential predictors [46]. We address both of these issues by applying the JD-R model to understand the specific causes and consequences of physician burnout using advanced SEM techniques.

### Limitations

It is important to interpret the results prudently since data emerges from a cross-sectional research design, which limits our ability to ascertain causality. A longitudinal approach with an objective measure of withdrawal (i.e. turnover) would lend further credence to the results. However, the fact that this population represents a challenge to partake in surveys makes it difficult to operationalize a repeated measurement approach. Another potential limitation concerns the fact that our results were obtained with self-report measures from a single source which might be confounded by common method variance. However, our analysis found that it was not a serious issue. Moreover, due to organizational restrictions, shortened scales were used and thus the full range of the focal constructs may not be effectively captured. Another potential limitation is that the full spectrum of propositions inherent to the JD-R model were not tested e.g. the ‘buffer effect’ and ‘coping effect’ hypotheses, and this is something that should be tested in future research. Nevertheless, in the present study, we were concerned with the application of the JD-R model to chart the effects of job demands and job resources on professional turnover intentions through intermediary mechanisms rather testing a fully-fledged model JD-R model (containing only specific proposition tests). Finally, it is quite possible that some of the physicians’ personality traits may play a role in the burnout process and they were not captured in the present study. Doing so may be especially important in light of recent research suggesting that individual factors such as personality can explain more variance in burnout than work environment factors [6]. Resilience, overinvestment in work, self-diagnosis, and the “God complex” are among the additional individual characteristics that may be worthy of exploration in future research [40].

### Practical implications

As job demands associated with the health impairment process are central to burnout among physicians and their desire to leave the medical profession, initiatives intended to optimize workload and stress are imperative. More specifically, it is essential that physicians have the requisite resources to deal with their overbearing workload. Some clinical and administrative responsibilities that hinder physicians’ ability to practice could be shared with other healthcare professionals, problem-solving mechanisms could be implemented to eliminate irritants, and forums could be created whereby physicians can express their psychological suffering [21]. More specifically, aside from informal meetings in the hallway, institutions could support physicians by offering to moderate discussion groups.

Given the importance of reducing workload and stress as well as ensuring physicians experience meaning at work, participatory and social support initiatives aimed at sharing responsibilities within teams may constitute a vital resource to help them cope [5].

For example, permitting other caregivers to defuse a conflict situation with patients may considerably reduce the pressure that physicians feel and reinforce the meaning of their work. Being able to regain emotional balance after having experienced difficult situations or those beyond one’s control is crucial. Supporting the suggestions above, prior research has found that a participatory action approach to work combined with social support groups (aimed at peer interaction to solve work related problems) is directly linked to reduced burnout among health care professionals in an intervention study [20]. Thus, these suggestions likely provide a viable platform to free up the excessive stress and workload experienced by physicians and ensure that they have the time necessary to invest in meaningful tasks. Above all, physicians must recognize their own vulnerability to stress, be vigilant for alarm signals and establish healthy limits between their work and their personal life [38]. A final practical implication stemming from the present investigation is that patient recognition should be fostered in the hospital context given its important role in alleviating burnout. More specifically, HR professionals and line managers can look at ways to stimulate patients to show their appreciation for high quality care provision such as informally acknowledging the work of physicians and devising thank you letters.

## Data Availability

The datasets used and analyzed during the current study are available from the corresponding author on reasonable request.
